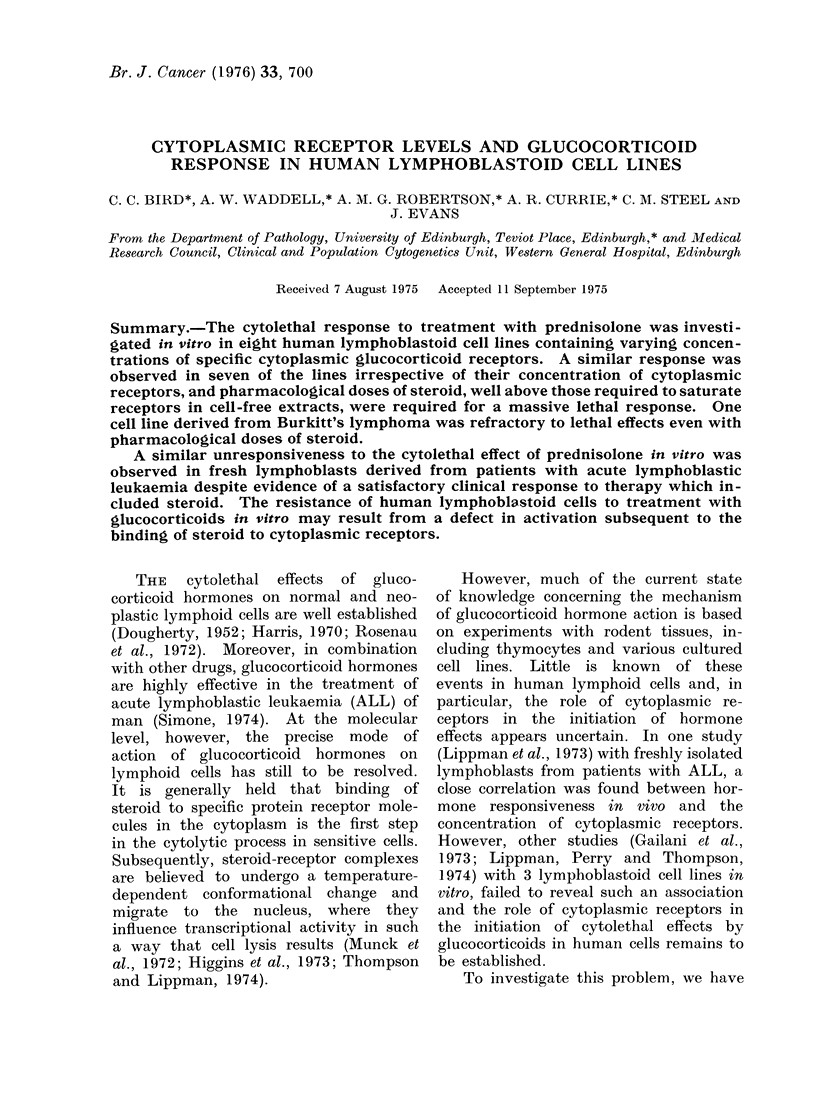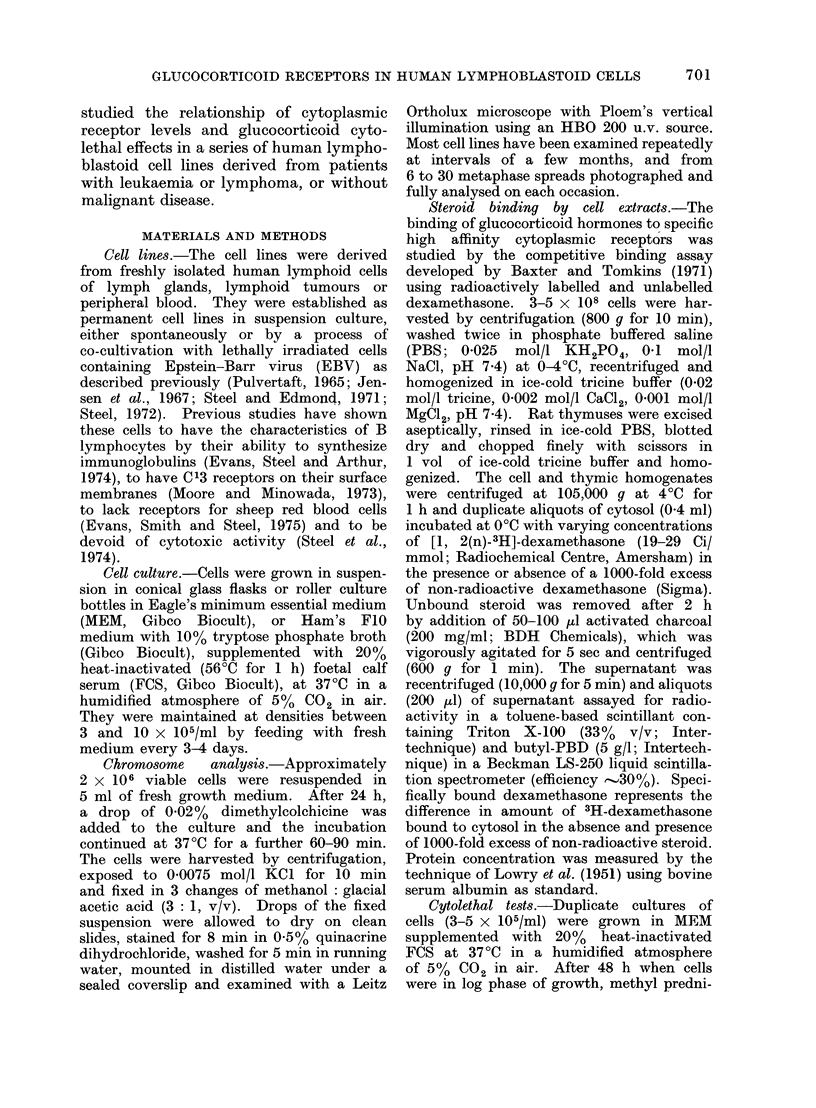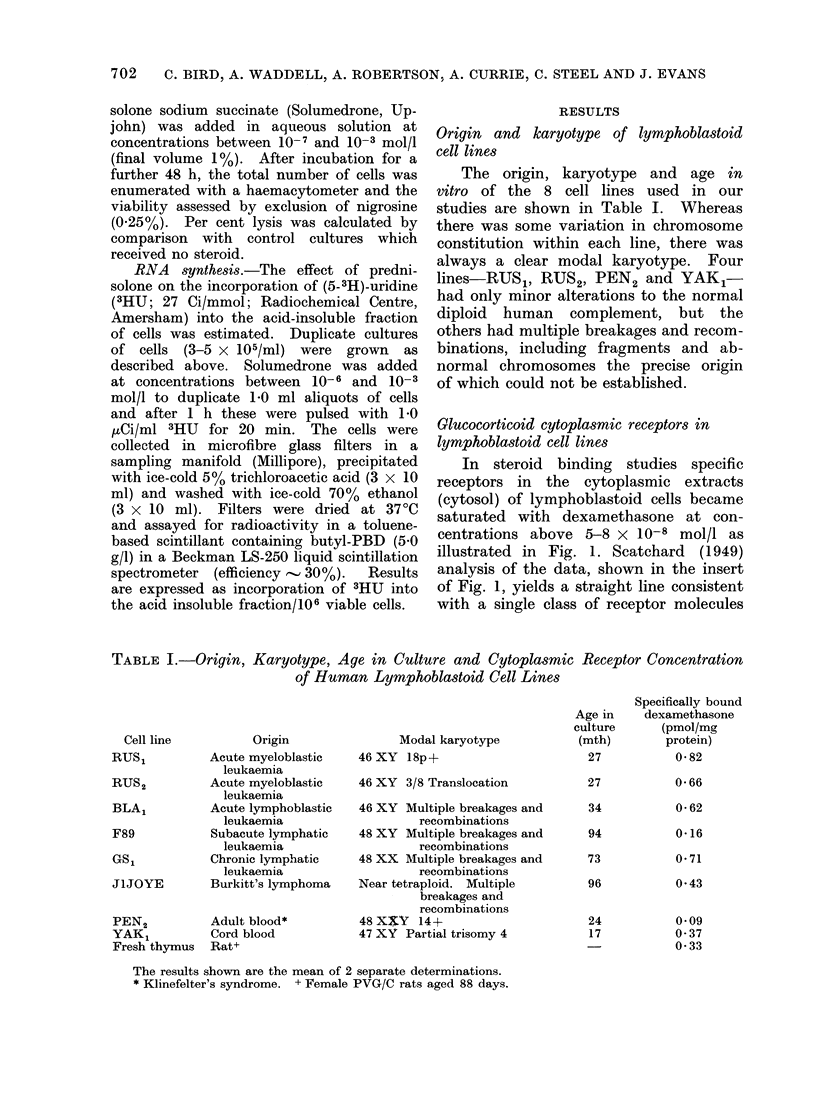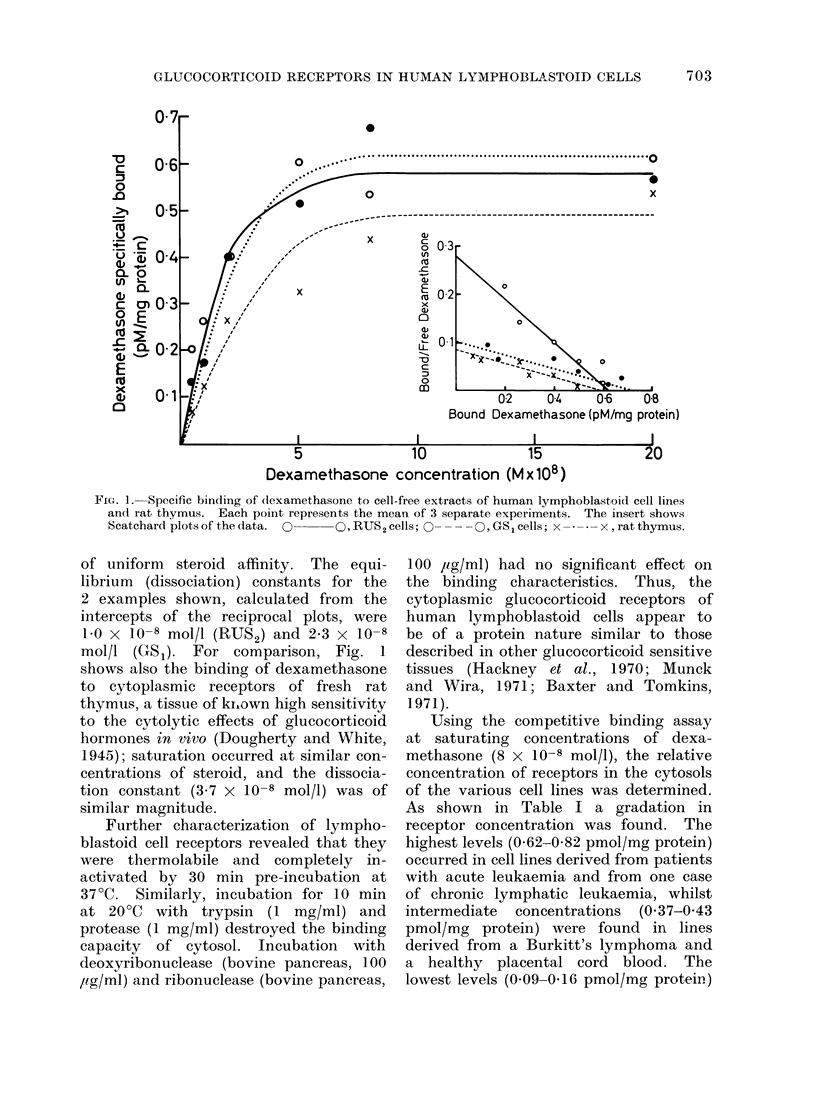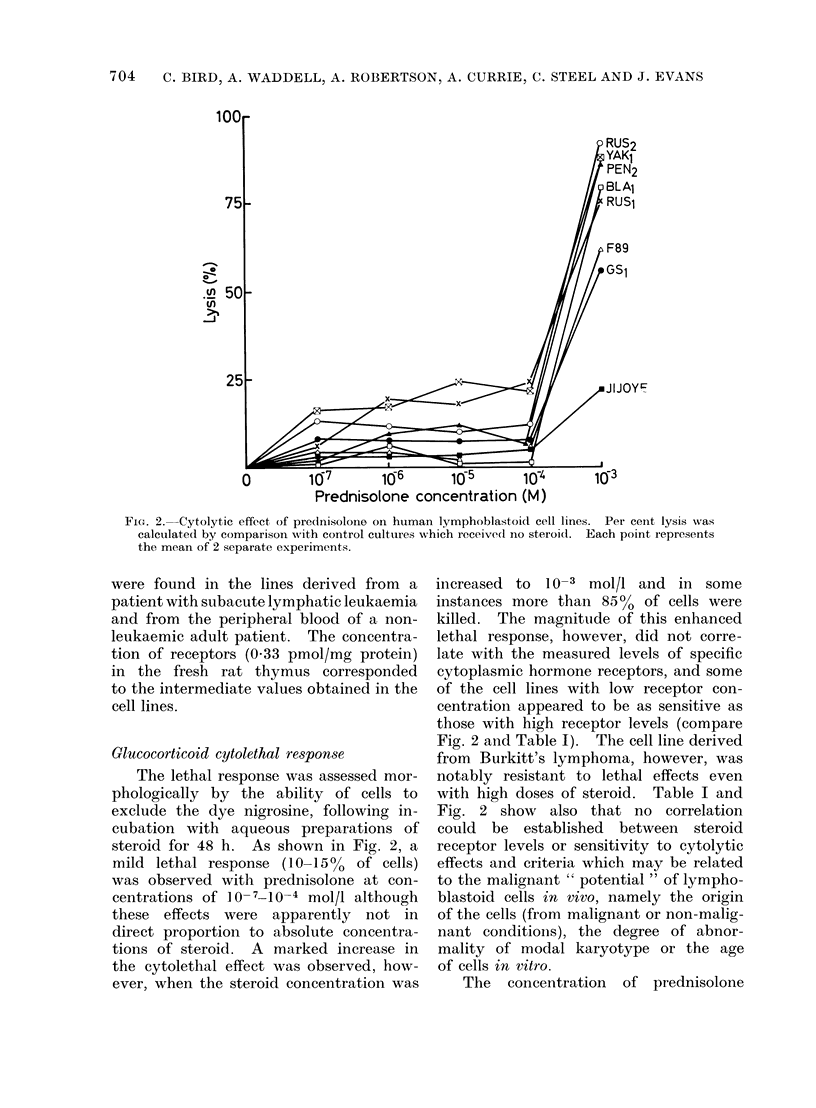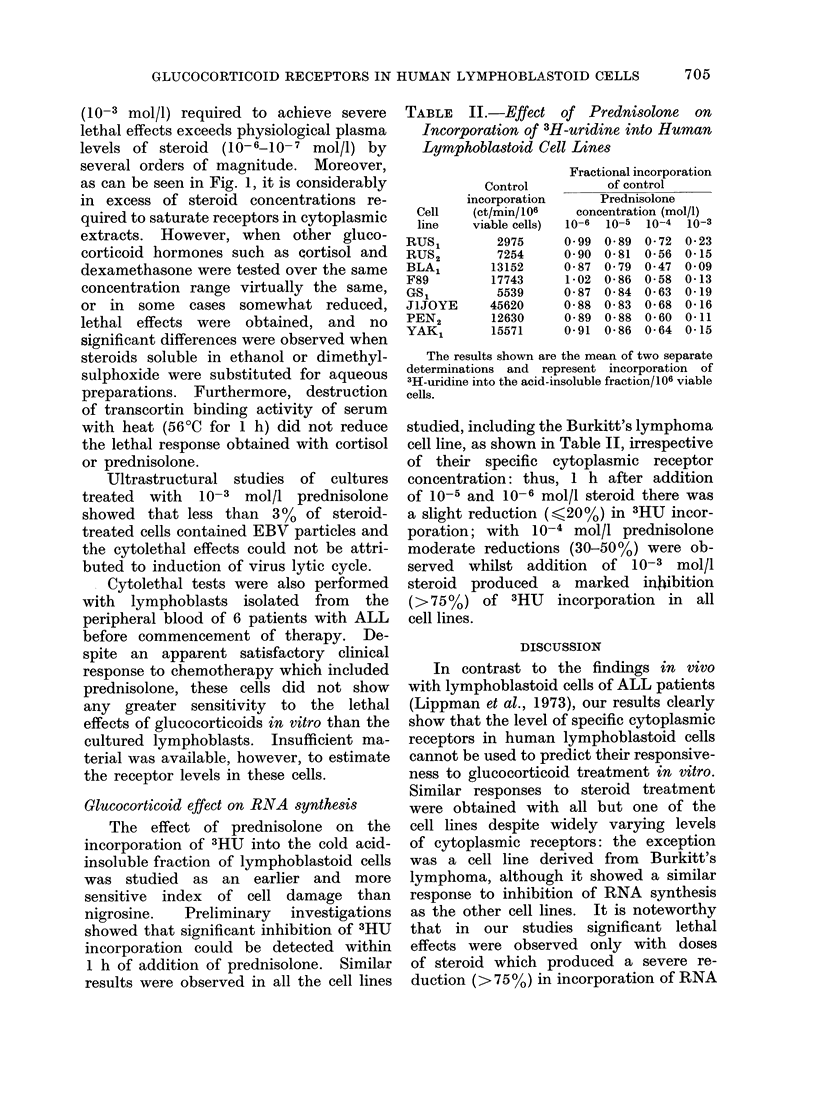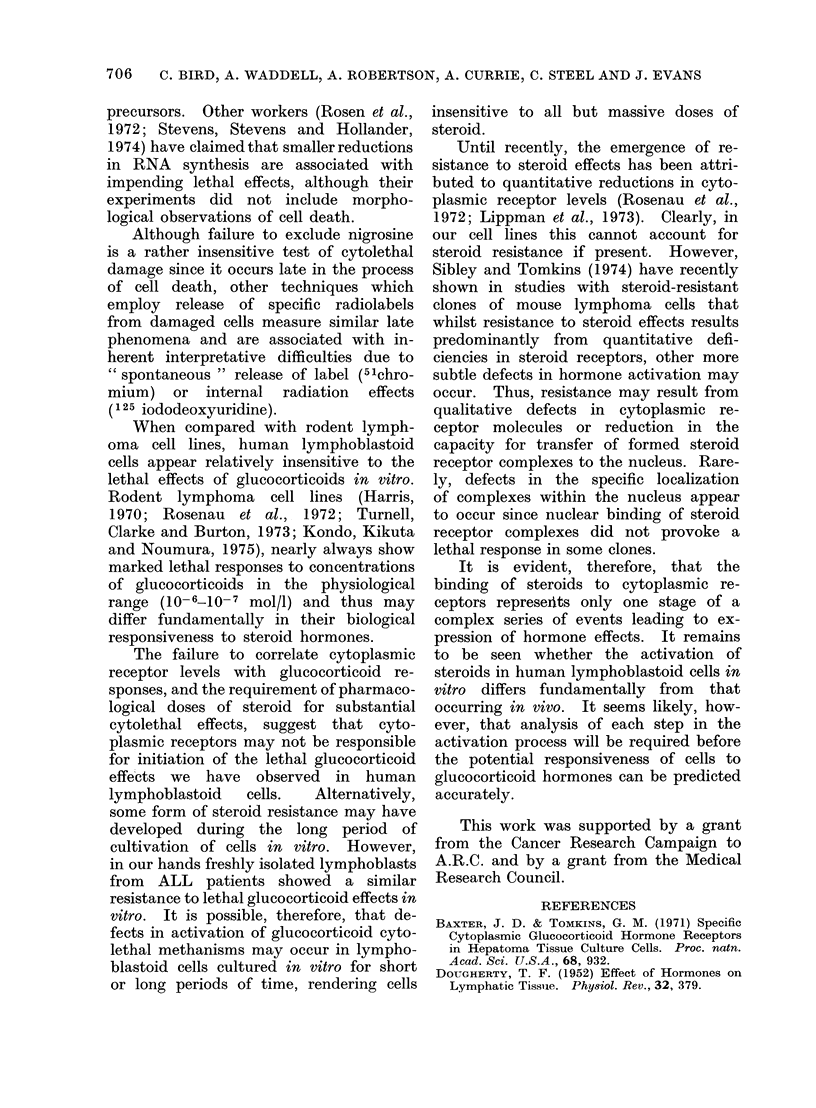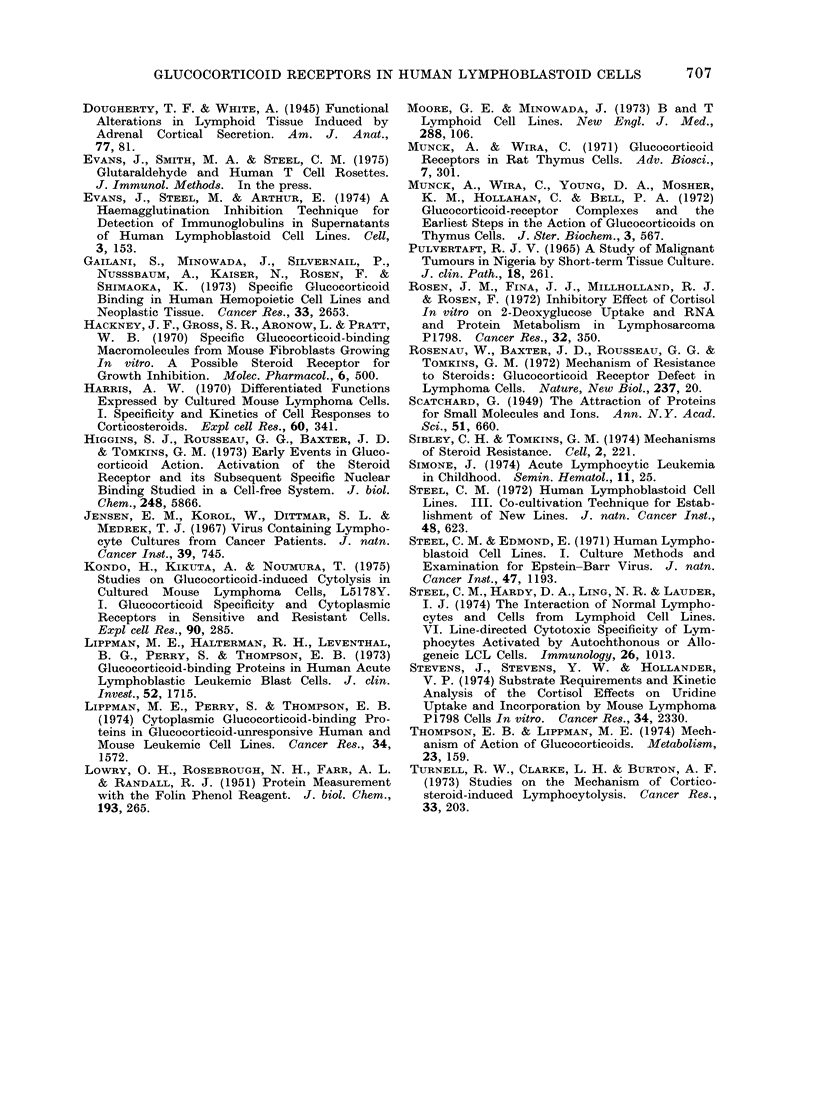# Cytoplasmic receptor levels and glucocorticoid response in human lymphoblastoid cell lines.

**DOI:** 10.1038/bjc.1975.281

**Published:** 1975-12

**Authors:** C. C. Bird, A. W. Waddell, A. M. Robertson, A. R. Currie, C. M. Steel, J. Evans

## Abstract

The cytolethal response to treatment with prednisolone was investigated in vitro in eight human lymphoblastoid cell lines containing varying concentrations of specific cytoplasmic glucocorticoid receptors. A similar response was observed in seven of the lines irrespective of their concentration of cytoplasmic receptors and pharmacological doses of steroid, well above those required to saturate receptors in cell-free extracts, were required for a massive lethal response. One cell line derived from Burkitt's lymphoma was refractory to lethal effects even with pharmacological doses of steroid. A similar unresponsiveness to the cytolethal effect of prednisolone in vitro was observed in fresh lymphoblasts derived from patients with acute lymphoblastic leukaemia despite evidence of a satisfactory clinical response to therapy which included steroid. The resistance of human lymphoblastoid cells to treatment with glucocorticoids in vitro may result from a defect in activation subsequent to the binding of steroid to cytoplasmic receptors.


*Br. J. Cancer* (1976) **33**, 700

### CYTOPLASMIC RECEPTOR LEVELS AND GLUCOCORTICOID RESPONSE IN HUMAN LYMPHOBLASTOID CELL LINES

C. C. BIRD*, A. W. WADDELL,* A. M. G. ROBERTSON,* A. R. CURRIE,* C. M. STEEL AND J. EVANS

*From the Department of Pathology, University of Edinburgh, Teviot Place, Edinburgh,* and Medical Research Council, Clinical and Population Cytogenetics Unit, Western General Hospital, Edinburgh*

Received 7 August 1975 Accepted 11 September 1975

**Summary.—The cytolethal response to treatment with prednisolone was investigated *in vitro* in eight human lymphoblastoid cell lines containing varying concentrations of specific cytoplasmic glucocorticoid receptors. A similar response was observed in seven of the lines irrespective of their concentration of cytoplasmic receptors, and pharmacological doses of steroid, well above those required to saturate receptors in cell-free extracts, were required for a massive lethal response. One cell line derived from Burkitt's lymphoma was refractory to lethal effects even with pharmacological doses of steroid.**

**A similar unresponsiveness to the cytolethal effect of prednisolone *in vitro* was observed in fresh lymphoblasts derived from patients with acute lymphoblastic leukaemia despite evidence of a satisfactory clinical response to therapy which included steroid. The resistance of human lymphoblastoid cells to treatment with glucocorticoids *in vitro* may result from a defect in activation subsequent to the binding of steroid to cytoplasmic receptors.**

THE cytolethal effects of glucocorticoid hormones on normal and neoplastic lymphoid cells are well established (Dougherty, 1952; Harris, 1970; Rosenau *et al.*, 1972). Moreover, in combination with other drugs, glucocorticoid hormones are highly effective in the treatment of acute lymphoblastic leukaemia (ALL) of man (Simone, 1974). At the molecular level, however, the precise mode of action of glucocorticoid hormones on lymphoid cells has still to be resolved. It is generally held that binding of steroid to specific protein receptor molecules in the cytoplasm is the first step in the cytolytic process in sensitive cells. Subsequently, steroid-receptor complexes are believed to undergo a temperature-dependent conformational change and migrate to the nucleus, where they influence transcriptional activity in such a way that cell lysis results (Munck *et al.*, 1972; Higgins *et al.*, 1973; Thompson and Lippman, 1974).

However, much of the current state of knowledge concerning the mechanism of glucocorticoid hormone action is based on experiments with rodent tissues, including thymocytes and various cultured cell lines. Little is known of these events in human lymphoid cells and, in particular, the role of cytoplasmic receptors in the initiation of hormone effects appears uncertain. In one study (Lippman *et al.*, 1973) with freshly isolated lymphoblasts from patients with ALL, a close correlation was found between hormone responsiveness *in vivo* and the concentration of cytoplasmic receptors. However, other studies (Gailani *et al.*, 1973; Lippman, Perry and Thompson, 1974) with 3 lymphoblastoid cell lines *in vitro*, failed to reveal such an association and the role of cytoplasmic receptors in the initiation of cytolethal effects by glucocorticoids in human cells remains to be established.

To investigate this problem, we have studied the relationship of cytoplasmic receptor levels and glucocorticoid cytolethal effects in a series of human lymphoblastoid cell lines derived from patients with leukaemia or lymphoma, or without malignant disease.

### MATERIALS AND METHODS

*Cell lines.*—The cell lines were derived from freshly isolated human lymphoid cells of lymph glands, lymphoid tumours or peripheral blood. They were established as permanent cell lines in suspension culture, either spontaneously or by a process of co-cultivation with lethally irradiated cells containing Epstein–Barr virus (EBV) as described previously (Pulvertaft, 1965; Jensen *et al.*, 1967; Steel and Edmond, 1971; Steel, 1972). Previous studies have shown these cells to have the characteristics of B lymphocytes by their ability to synthesize immunoglobulins (Evans, Steel and Arthur, 1974), to have C13 receptors on their surface membranes (Moore and Minowada, 1973), to lack receptors for sheep red blood cells (Evans, Smith and Steel, 1975) and to be devoid of cytotoxic activity (Steel *et al.*, 1974).

*Cell culture.*—Cells were grown in suspension in conical glass flasks or roller culture bottles in Eagle's minimum essential medium (MEM, Gibco Biocult), or Ham's F10 medium with 10% tryptose phosphate broth (Gibco Biocult), supplemented with 20% heat-inactivated (56°C for 1 h) foetal calf serum (FCS, Gibco Biocult), at 37°C in a humidified atmosphere of 5% $CO_2$ in air. They were maintained at densities between 3 and $10 \times 10^5$/ml by feeding with fresh medium every 3–4 days.

*Chromosome analysis.*—Approximately $2 \times 10^6$ viable cells were resuspended in 5 ml of fresh growth medium. After 24 h, a drop of 0·02% dimethylcolchicine was added to the culture and the incubation continued at 37°C for a further 60–90 min. The cells were harvested by centrifugation, exposed to 0·0075 mol/l KCl for 10 min and fixed in 3 changes of methanol : glacial acetic acid (3 : 1, v/v). Drops of the fixed suspension were allowed to dry on clean slides, stained for 8 min in 0·5% quinacrine dihydrochloride, washed for 5 min in running water, mounted in distilled water under a sealed coverslip and examined with a Leitz Ortholux microscope with Ploem's vertical illumination using an HBO 200 u.v. source. Most cell lines have been examined repeatedly at intervals of a few months, and from 6 to 30 metaphase spreads photographed and fully analysed on each occasion.

*Steroid binding by cell extracts.*—The binding of glucocorticoid hormones to specific high affinity cytoplasmic receptors was studied by the competitive binding assay developed by Baxter and Tomkins (1971) using radioactively labelled and unlabelled dexamethasone. $3–5 \times 10^8$ cells were harvested by centrifugation (800 *g* for 10 min), washed twice in phosphate buffered saline (PBS; 0·025 mol/l $KH_2PO_4$, 0·1 mol/l NaCl, pH 7·4) at 0–4°C, recentrifuged and homogenized in ice-cold tricine buffer (0·02 mol/l tricine, 0·002 mol/l $CaCl_2$, 0·001 mol/l $MgCl_2$, pH 7·4). Rat thymuses were excised aseptically, rinsed in ice-cold PBS, blotted dry and chopped finely with scissors in 1 vol of ice-cold tricine buffer and homogenized. The cell and thymic homogenates were centrifuged at 105,000 *g* at 4°C for 1 h and duplicate aliquots of cytosol (0·4 ml) incubated at 0°C with varying concentrations of [1, 2(n)-$^3$H]-dexamethasone (19–29 Ci/mmol; Radiochemical Centre, Amersham) in the presence or absence of a 1000-fold excess of non-radioactive dexamethasone (Sigma). Unbound steroid was removed after 2 h by addition of 50–100 $\mu$l activated charcoal (200 mg/ml; BDH Chemicals), which was vigorously agitated for 5 sec and centrifuged (600 *g* for 1 min). The supernatant was recentrifuged (10,000 *g* for 5 min) and aliquots (200 $\mu$l) of supernatant assayed for radioactivity in a toluene-based scintillant containing Triton X-100 (33% v/v; Intertechnique) and butyl-PBD (5 g/l; Intertechnique) in a Beckman LS-250 liquid scintillation spectrometer (efficiency ~30%). Specifically bound dexamethasone represents the difference in amount of $^3$H-dexamethasone bound to cytosol in the absence and presence of 1000-fold excess of non-radioactive steroid. Protein concentration was measured by the technique of Lowry *et al.* (1951) using bovine serum albumin as standard.

*Cytolethal tests.*—Duplicate cultures of cells ($3–5 \times 10^5$/ml) were grown in MEM supplemented with 20% heat-inactivated FCS at 37°C in a humidified atmosphere of 5% $CO_2$ in air. After 48 h when cells were in log phase of growth, methyl prednisolone sodium succinate (Solumedrone, Upjohn) was added in aqueous solution at concentrations between $10^{-7}$ and $10^{-3}$ mol/l (final volume 1%). After incubation for a further 48 h, the total number of cells was enumerated with a haemacytometer and the viability assessed by exclusion of nigrosine (0·25%). Per cent lysis was calculated by comparison with control cultures which received no steroid.

*RNA synthesis.*—The effect of prednisolone on the incorporation of (5-$^3$H)-uridine ($^3$HU; 27 Ci/mmol; Radiochemical Centre, Amersham) into the acid-insoluble fraction of cells was estimated. Duplicate cultures of cells ($3–5 \times 10^5$/ml) were grown as described above. Solumedrone was added at concentrations between $10^{-6}$ and $10^{-3}$ mol/l to duplicate 1·0 ml aliquots of cells and after 1 h these were pulsed with 1·0 $\mu$Ci/ml $^3$HU for 20 min. The cells were collected in microfibre glass filters in a sampling manifold (Millipore), precipitated with ice-cold 5% trichloroacetic acid ($3 \times 10$ ml) and washed with ice-cold 70% ethanol ($3 \times 10$ ml). Filters were dried at 37°C and assayed for radioactivity in a toluene-based scintillant containing butyl-PBD (5·0 g/l) in a Beckman LS-250 liquid scintillation spectrometer (efficiency $\sim$ 30%). Results are expressed as incorporation of $^3$HU into the acid insoluble fraction/$10^6$ viable cells.

### RESULTS

### *Origin and karyotype of lymphoblastoid cell lines*

The origin, karyotype and age *in vitro* of the 8 cell lines used in our studies are shown in Table I. Whereas there was some variation in chromosome constitution within each line, there was always a clear modal karyotype. Four lines—$RUS_1$, $RUS_2$, $PEN_2$ and $YAK_1$—had only minor alterations to the normal diploid human complement, but the others had multiple breakages and recombinations, including fragments and abnormal chromosomes the precise origin of which could not be established.

### *Glucocorticoid cytoplasmic receptors in lymphoblastoid cell lines*

In steroid binding studies specific receptors in the cytoplasmic extracts (cytosol) of lymphoblastoid cells became saturated with dexamethasone at concentrations above $5–8 \times 10^{-8}$ mol/l as illustrated in Fig. 1. Scatchard (1949) analysis of the data, shown in the insert of Fig. 1, yields a straight line consistent with a single class of receptor molecules

TABLE I.—*Origin, Karyotype, Age in Culture and Cytoplasmic Receptor Concentration of Human Lymphoblastoid Cell Lines*

| Cell line | Origin | Modal karyotype | Age in culture (mth) | Specifically bound dexamethasone (pmol/mg protein) |
|---|---|---|---|---|
| $RUS_1$ | Acute myeloblastic leukaemia | 46 XY 18p+ | 27 | 0·82 |
| $RUS_2$ | Acute myeloblastic leukaemia | 46 XY 3/8 Translocation | 27 | 0·66 |
| $BLA_1$ | Acute lymphoblastic leukaemia | 46 XY Multiple breakages and recombinations | 34 | 0·62 |
| F89 | Subacute lymphatic leukaemia | 48 XY Multiple breakages and recombinations | 94 | 0·16 |
| $GS_1$ | Chronic lymphatic leukaemia | 48 XX Multiple breakages and recombinations | 73 | 0·71 |
| J1JOYE | Burkitt's lymphoma | Near tetraploid. Multiple breakages and recombinations | 96 | 0·43 |
| $PEN_2$ | Adult blood* | 48 XXY 14+ | 24 | 0·09 |
| $YAK_1$ | Cord blood | 47 XY Partial trisomy 4 | 17 | 0·37 |
| Fresh thymus | Rat+ | | — | 0·33 |

The results shown are the mean of 2 separate determinations.
* Klinefelter's syndrome. + Female PVG/C rats aged 88 days.

FIG. 1.—Specific binding of dexamethasone to cell-free extracts of human lymphoblastoid cell lines and rat thymus. Each point represents the mean of 3 separate experiments. The insert shows Scatchard plots of the data. ○———○, $RUS_2$ cells; ○– – – –○, $GS_1$ cells; ×–·–·–×, rat thymus.

of uniform steroid affinity. The equilibrium (dissociation) constants for the 2 examples shown, calculated from the intercepts of the reciprocal plots, were $1{\cdot}0 \times 10^{-8}$ mol/l ($RUS_2$) and $2{\cdot}3 \times 10^{-8}$ mol/l ($GS_1$). For comparison, Fig. 1 shows also the binding of dexamethasone to cytoplasmic receptors of fresh rat thymus, a tissue of known high sensitivity to the cytolytic effects of glucocorticoid hormones *in vivo* (Dougherty and White, 1945); saturation occurred at similar concentrations of steroid, and the dissociation constant ($3{\cdot}7 \times 10^{-8}$ mol/l) was of similar magnitude.

Further characterization of lymphoblastoid cell receptors revealed that they were thermolabile and completely inactivated by 30 min pre-incubation at 37°C. Similarly, incubation for 10 min at 20°C with trypsin (1 mg/ml) and protease (1 mg/ml) destroyed the binding capacity of cytosol. Incubation with deoxyribonuclease (bovine pancreas, 100 μg/ml) and ribonuclease (bovine pancreas, 100 μg/ml) had no significant effect on the binding characteristics. Thus, the cytoplasmic glucocorticoid receptors of human lymphoblastoid cells appear to be of a protein nature similar to those described in other glucocorticoid sensitive tissues (Hackney *et al.*, 1970; Munck and Wira, 1971; Baxter and Tomkins, 1971).

Using the competitive binding assay at saturating concentrations of dexamethasone ($8 \times 10^{-8}$ mol/l), the relative concentration of receptors in the cytosols of the various cell lines was determined. As shown in Table I a gradation in receptor concentration was found. The highest levels (0·62–0·82 pmol/mg protein) occurred in cell lines derived from patients with acute leukaemia and from one case of chronic lymphatic leukaemia, whilst intermediate concentrations (0·37–0·43 pmol/mg protein) were found in lines derived from a Burkitt's lymphoma and a healthy placental cord blood. The lowest levels (0·09–0·16 pmol/mg protein)

FIG. 2.—Cytolytic effect of prednisolone on human lymphoblastoid cell lines. Per cent lysis was calculated by comparison with control cultures which received no steroid. Each point represents the mean of 2 separate experiments.

were found in the lines derived from a patient with subacute lymphatic leukaemia and from the peripheral blood of a non-leukaemic adult patient. The concentration of receptors (0·33 pmol/mg protein) in the fresh rat thymus corresponded to the intermediate values obtained in the cell lines.

*Glucocorticoid cytolethal response*

The lethal response was assessed morphologically by the ability of cells to exclude the dye nigrosine, following incubation with aqueous preparations of steroid for 48 h. As shown in Fig. 2, a mild lethal response (10–15% of cells) was observed with prednisolone at concentrations of $10^{-7}$–$10^{-4}$ mol/l although these effects were apparently not in direct proportion to absolute concentrations of steroid. A marked increase in the cytolethal effect was observed, however, when the steroid concentration was increased to $10^{-3}$ mol/l and in some instances more than 85% of cells were killed. The magnitude of this enhanced lethal response, however, did not correlate with the measured levels of specific cytoplasmic hormone receptors, and some of the cell lines with low receptor concentration appeared to be as sensitive as those with high receptor levels (compare Fig. 2 and Table I). The cell line derived from Burkitt's lymphoma, however, was notably resistant to lethal effects even with high doses of steroid. Table I and Fig. 2 show also that no correlation could be established between steroid receptor levels or sensitivity to cytolytic effects and criteria which may be related to the malignant " potential " of lymphoblastoid cells *in vivo*, namely the origin of the cells (from malignant or non-malignant conditions), the degree of abnormality of modal karyotype or the age of cells *in vitro*.

The concentration of prednisolone

($10^{-3}$ mol/l) required to achieve severe lethal effects exceeds physiological plasma levels of steroid ($10^{-6}$–$10^{-7}$ mol/l) by several orders of magnitude. Moreover, as can be seen in Fig. 1, it is considerably in excess of steroid concentrations required to saturate receptors in cytoplasmic extracts. However, when other glucocorticoid hormones such as cortisol and dexamethasone were tested over the same concentration range virtually the same, or in some cases somewhat reduced, lethal effects were obtained, and no significant differences were observed when steroids soluble in ethanol or dimethylsulphoxide were substituted for aqueous preparations. Furthermore, destruction of transcortin binding activity of serum with heat (56°C for 1 h) did not reduce the lethal response obtained with cortisol or prednisolone.

Ultrastructural studies of cultures treated with $10^{-3}$ mol/l prednisolone showed that less than 3% of steroid-treated cells contained EBV particles and the cytolethal effects could not be attributed to induction of virus lytic cycle.

Cytolethal tests were also performed with lymphoblasts isolated from the peripheral blood of 6 patients with ALL before commencement of therapy. Despite an apparent satisfactory clinical response to chemotherapy which included prednisolone, these cells did not show any greater sensitivity to the lethal effects of glucocorticoids *in vitro* than the cultured lymphoblasts. Insufficient material was available, however, to estimate the receptor levels in these cells.

### *Glucocorticoid effect on RNA synthesis*

The effect of prednisolone on the incorporation of $^3$HU into the cold acid-insoluble fraction of lymphoblastoid cells was studied as an earlier and more sensitive index of cell damage than nigrosine. Preliminary investigations showed that significant inhibition of $^3$HU incorporation could be detected within 1 h of addition of prednisolone. Similar results were observed in all the cell lines

TABLE II.—*Effect of Prednisolone on Incorporation of $^3$H-uridine into Human Lymphoblastoid Cell Lines*

| Cell line | Control incorporation (ct/min/$10^6$ viable cells) | Fractional incorporation of control — Prednisolone concentration (mol/l) $10^{-6}$ | $10^{-5}$ | $10^{-4}$ | $10^{-3}$ |
|---|---|---|---|---|---|
| $RUS_1$ | 2975 | 0·99 | 0·89 | 0·72 | 0·23 |
| $RUS_2$ | 7254 | 0·90 | 0·81 | 0·56 | 0·15 |
| $BLA_1$ | 13152 | 0·87 | 0·79 | 0·47 | 0·09 |
| F89 | 17743 | 1·02 | 0·86 | 0·58 | 0·13 |
| $GS_1$ | 5539 | 0·87 | 0·84 | 0·63 | 0·19 |
| J1JOYE | 45620 | 0·88 | 0·83 | 0·68 | 0·16 |
| $PEN_2$ | 12630 | 0·89 | 0·88 | 0·60 | 0·11 |
| $YAK_1$ | 15571 | 0·91 | 0·86 | 0·64 | 0·15 |

The results shown are the mean of two separate determinations and represent incorporation of $^3$H-uridine into the acid-insoluble fraction/$10^6$ viable cells.

studied, including the Burkitt's lymphoma cell line, as shown in Table II, irrespective of their specific cytoplasmic receptor concentration: thus, 1 h after addition of $10^{-5}$ and $10^{-6}$ mol/l steroid there was a slight reduction ($\leq$20%) in $^3$HU incorporation; with $10^{-4}$ mol/l prednisolone moderate reductions (30–50%) were observed whilst addition of $10^{-3}$ mol/l steroid produced a marked inhibition (>75%) of $^3$HU incorporation in all cell lines.

### DISCUSSION

In contrast to the findings *in vivo* with lymphoblastoid cells of ALL patients (Lippman *et al.*, 1973), our results clearly show that the level of specific cytoplasmic receptors in human lymphoblastoid cells cannot be used to predict their responsiveness to glucocorticoid treatment *in vitro*. Similar responses to steroid treatment were obtained with all but one of the cell lines despite widely varying levels of cytoplasmic receptors: the exception was a cell line derived from Burkitt's lymphoma, although it showed a similar response to inhibition of RNA synthesis as the other cell lines. It is noteworthy that in our studies significant lethal effects were observed only with doses of steroid which produced a severe reduction (>75%) in incorporation of RNA

precursors. Other workers (Rosen *et al.*, 1972; Stevens, Stevens and Hollander, 1974) have claimed that smaller reductions in RNA synthesis are associated with impending lethal effects, although their experiments did not include morphological observations of cell death.

Although failure to exclude nigrosine is a rather insensitive test of cytolethal damage since it occurs late in the process of cell death, other techniques which employ release of specific radiolabels from damaged cells measure similar late phenomena and are associated with inherent interpretative difficulties due to " spontaneous " release of label ($^{51}$chromium) or internal radiation effects ($^{125}$ iododeoxyuridine).

When compared with rodent lymphoma cell lines, human lymphoblastoid cells appear relatively insensitive to the lethal effects of glucocorticoids *in vitro*. Rodent lymphoma cell lines (Harris, 1970; Rosenau *et al.*, 1972; Turnell, Clarke and Burton, 1973; Kondo, Kikuta and Noumura, 1975), nearly always show marked lethal responses to concentrations of glucocorticoids in the physiological range ($10^{-6}$–$10^{-7}$ mol/l) and thus may differ fundamentally in their biological responsiveness to steroid hormones.

The failure to correlate cytoplasmic receptor levels with glucocorticoid responses, and the requirement of pharmacological doses of steroid for substantial cytolethal effects, suggest that cytoplasmic receptors may not be responsible for initiation of the lethal glucocorticoid effects we have observed in human lymphoblastoid cells. Alternatively, some form of steroid resistance may have developed during the long period of cultivation of cells *in vitro*. However, in our hands freshly isolated lymphoblasts from ALL patients showed a similar resistance to lethal glucocorticoid effects *in vitro*. It is possible, therefore, that defects in activation of glucocorticoid cytolethal methanisms may occur in lymphoblastoid cells cultured *in vitro* for short or long periods of time, rendering cells insensitive to all but massive doses of steroid.

Until recently, the emergence of resistance to steroid effects has been attributed to quantitative reductions in cytoplasmic receptor levels (Rosenau *et al.*, 1972; Lippman *et al.*, 1973). Clearly, in our cell lines this cannot account for steroid resistance if present. However, Sibley and Tomkins (1974) have recently shown in studies with steroid-resistant clones of mouse lymphoma cells that whilst resistance to steroid effects results predominantly from quantitative deficiencies in steroid receptors, other more subtle defects in hormone activation may occur. Thus, resistance may result from qualitative defects in cytoplasmic receptor molecules or reduction in the capacity for transfer of formed steroid receptor complexes to the nucleus. Rarely, defects in the specific localization of complexes within the nucleus appear to occur since nuclear binding of steroid receptor complexes did not provoke a lethal response in some clones.

It is evident, therefore, that the binding of steroids to cytoplasmic receptors represents only one stage of a complex series of events leading to expression of hormone effects. It remains to be seen whether the activation of steroids in human lymphoblastoid cells *in vitro* differs fundamentally from that occurring *in vivo*. It seems likely, however, that analysis of each step in the activation process will be required before the potential responsiveness of cells to glucocorticoid hormones can be predicted accurately.

This work was supported by a grant from the Cancer Research Campaign to A.R.C. and by a grant from the Medical Research Council.

REFERENCES

BAXTER, J. D. & TOMKINS, G. M. (1971) Specific Cytoplasmic Glucocorticoid Hormone Receptors in Hepatoma Tissue Culture Cells. *Proc. natn. Acad. Sci. U.S.A.*, **68**, 932.

DOUGHERTY, T. F. (1952) Effect of Hormones on Lymphatic Tissue. *Physiol. Rev.*, **32**, 379.

DOUGHERTY, T. F. & WHITE, A. (1945) Functional Alterations in Lymphoid Tissue Induced by Adrenal Cortical Secretion. *Am. J. Anat.*, **77**, 81.

EVANS, J., SMITH, M. A. & STEEL, C. M. (1975) Glutaraldehyde and Human T Cell Rosettes. *J. Immunol. Methods.* In the press.

EVANS, J., STEEL, M. & ARTHUR, E. (1974) A Haemagglutination Inhibition Technique for Detection of Immunoglobulins in Supernatants of Human Lymphoblastoid Cell Lines. *Cell*, **3**, 153.

GAILANI, S., MINOWADA, J., SILVERNAIL, P., NUSSSBAUM, A., KAISER, N., ROSEN, F. & SHIMAOKA, K. (1973) Specific Glucocorticoid Binding in Human Hemopoietic Cell Lines and Neoplastic Tissue. *Cancer Res.*, **33**, 2653.

HACKNEY, J. F., GROSS, S. R., ARONOW, L. & PRATT, W. B. (1970) Specific Glucocorticoid-binding Macromolecules from Mouse Fibroblasts Growing *In vitro*. A Possible Steroid Receptor for Growth Inhibition. *Molec. Pharmacol.*, **6**, 500.

HARRIS, A. W. (1970) Differentiated Functions Expressed by Cultured Mouse Lymphoma Cells. I. Specificity and Kinetics of Cell Responses to Corticosteroids. *Expl cell Res.*, **60**, 341.

HIGGINS, S. J., ROUSSEAU, G. G., BAXTER, J. D. & TOMKINS, G. M. (1973) Early Events in Glucocorticoid Action. Activation of the Steroid Receptor and its Subsequent Specific Nuclear Binding Studied in a Cell-free System. *J. biol. Chem.*, **248**, 5866.

JENSEN, E. M., KOROL, W., DITTMAR, S. L. & MEDREK, T. J. (1967) Virus Containing Lymphocyte Cultures from Cancer Patients. *J. natn. Cancer Inst.*, **39**, 745.

KONDO, H., KIKUTA, A. & NOUMURA, T. (1975) Studies on Glucocorticoid-induced Cytolysis in Cultured Mouse Lymphoma Cells, L5178Y. I. Glucocorticoid Specificity and Cytoplasmic Receptors in Sensitive and Resistant Cells. *Expl cell Res.*, **90**, 285.

LIPPMAN, M. E., HALTERMAN, R. H., LEVENTHAL, B. G., PERRY, S. & THOMPSON, E. B. (1973) Glucocorticoid-binding Proteins in Human Acute Lymphoblastic Leukemic Blast Cells. *J. clin. Invest.*, **52**, 1715.

LIPPMAN, M. E., PERRY, S. & THOMPSON, E. B. (1974) Cytoplasmic Glucocorticoid-binding Proteins in Glucocorticoid-unresponsive Human and Mouse Leukemic Cell Lines. *Cancer Res.*, **34**, 1572.

LOWRY, O. H., ROSEBROUGH, N. H., FARR, A. L. & RANDALL, R. J. (1951) Protein Measurement with the Folin Phenol Reagent. *J. biol. Chem.*, **193**, 265.

MOORE, G. E. & MINOWADA, J. (1973) B and T Lymphoid Cell Lines. *New Engl. J. Med.*, **288**, 106.

MUNCK, A. & WIRA, C. (1971) Glucocorticoid Receptors in Rat Thymus Cells. *Adv. Biosci.*, **7**, 301.

MUNCK, A., WIRA, C., YOUNG, D. A., MOSHER, K. M., HOLLAHAN, C. & BELL, P. A. (1972) Glucocorticoid-receptor Complexes and the Earliest Steps in the Action of Glucocorticoids on Thymus Cells. *J. Ster. Biochem.*, **3**, 567.

PULVERTAFT, R. J. V. (1965) A Study of Malignant Tumours in Nigeria by Short-term Tissue Culture. *J. clin. Path.*, **18**, 261.

ROSEN, J. M., FINA, J. J., MILLHOLLAND, R. J. & ROSEN, F. (1972) Inhibitory Effect of Cortisol *In vitro* on 2-Deoxyglucose Uptake and RNA and Protein Metabolism in Lymphosarcoma P1798. *Cancer Res.*, **32**, 350.

ROSENAU, W., BAXTER, J. D., ROUSSEAU, G. G. & TOMKINS, G. M. (1972) Mechanism of Resistance to Steroids: Glucocorticoid Receptor Defect in Lymphoma Cells. *Nature, New Biol.*, **237**, 20.

SCATCHARD, G. (1949) The Attraction of Proteins for Small Molecules and Ions. *Ann. N.Y. Acad. Sci.*, **51**, 660.

SIBLEY, C. H. & TOMKINS, G. M. (1974) Mechanisms of Steroid Resistance. *Cell*, **2**, 221.

SIMONE, J. (1974) Acute Lymphocytic Leukemia in Childhood. *Semin. Hematol.*, **11**, 25.

STEEL, C. M. (1972) Human Lymphoblastoid Cell Lines. III. Co-cultivation Technique for Establishment of New Lines. *J. natn. Cancer Inst.*, **48**, 623.

STEEL, C. M. & EDMOND, E. (1971) Human Lymphoblastoid Cell Lines. I. Culture Methods and Examination for Epstein–Barr Virus. *J. natn. Cancer Inst.*, **47**, 1193.

STEEL, C. M., HARDY, D. A., LING, N. R. & LAUDER, I. J. (1974) The Interaction of Normal Lymphocytes and Cells from Lymphoid Cell Lines. VI. Line-directed Cytotoxic Specificity of Lymphocytes Activated by Autochthonous or Allogeneic LCL Cells. *Immunology*, **26**, 1013.

STEVENS, J., STEVENS, Y. W. & HOLLANDER, V. P. (1974) Substrate Requirements and Kinetic Analysis of the Cortisol Effects on Uridine Uptake and Incorporation by Mouse Lymphoma P1798 Cells *In vitro*. *Cancer Res.*, **34**, 2330.

THOMPSON, E. B. & LIPPMAN, M. E. (1974) Mechanism of Action of Glucocorticoids. *Metabolism*, **23**, 159.

TURNELL, R. W., CLARKE, L. H. & BURTON, A. F. (1973) Studies on the Mechanism of Corticosteroid-induced Lymphocytolysis. *Cancer Res.*, **33**, 203.